# Physiological and molecular mechanisms underlying the quality changes of lotus seeds during different ripening periods

**DOI:** 10.3389/fpls.2026.1797105

**Published:** 2026-04-16

**Authors:** Weiting Chen, Shengchun Yang, YanChun Song, Meijiao Chen, Linshan Zhong, Fengqin Yao, Yinghui Wei, Zhifeng Wu, Fazhuang Lin, Yinhui Qiu

**Affiliations:** 1 Sanming Academy of Agricultural Sciences/Fujian Key Laboratory of Crop Genetic Improvement and Innovative Utilization for Mountain Area, Sha Xian, Fujian, China; 2 Fujian Jianning Lotus Seed Science Research Institute, Jian Ning, Fujian, China; 3 Luzhou Modern Agricultural Development Promotion Center, Luzhou, Sichuan, China; 4 The Soil Fertilizer Technical Station of Longyan City, Longyan, Fujian, China

**Keywords:** fruit maturity, lotus (*Nelumbo nucifera*), metabolomics, transcriptome, VOCs

## Abstract

Lotus seeds, which are renowned for their unique flavor and medicinal value, have become an important economic crop. Determining the optimal harvesting period is crucial for obtaining high-quality lotus seeds. From the perspectives of appearance and nutritional composition, we described the variations in lotus seeds “Jianxuan 30” during different developmental stages. Furthermore, we employed headspace solid-phase microextraction–gas chromatography–mass spectrometry (HS–SPME–GC–MS) and a transcriptomic analysis to identify the characteristic volatile metabolites and differentially expressed genes in mature lotus seeds. Our findings revealed that the morphological development of lotus seeds is not synchronized with the accumulation of nutrients. During the 15–20-day developmental period, although the rate of morphological change slows, the contents and weights of nutrients increase rapidly. Upon maturation, hexadecanoic acid methyl ester, 1-butanol, 3-methylbutanal, and methylparinarate are released, and their biosynthesis is attributed to the transcriptional activation of lipoxygenase (LOX), alcohol dehydrogenase (ADH), and aldehyde dehydrogenase (ALDH). Consequently, relying on visual appearance to assess the ripeness of lotus seeds is inaccurate. We elucidated the key volatile metabolites produced during the maturation of lotus seeds and the marker genes that promote their biosynthesis, laying a molecular foundation for future applications of Odor scanner to determine the optimal harvesting period for lotus seeds.

## Introduction

Lotus (*Nelumbo nucifera* Gaertn), an important aquatic plant, is widely consumed and cultivated in China and other Asian countries because of its nutritional and medicinal value (Jia et al., 2026). Lotus seeds and lotus roots are important components of lotus that are harvested; mature lotus seeds are not only rich in starch, high-quality protein, dietary fiber, vitamin C and mineral elements such as calcium, magnesium, and zinc but also contain secondary metabolites such as polyphenols, flavonoids, and alkaloids that possess antioxidant, lipid-lowering, and immunomodulatory functions (Muzalfa et al., 2021). The quality of lotus seeds is crucial to their commercial value, and choosing the appropriate harvesting period can ensure the quality of lotus seeds. Therefore, analyzing the maturation characteristics of lotus seeds is necessary.

When fruits reach maturity, their appearance and texture undergo corresponding transformations (Zhixia et al., 2024). These characteristics, when combined with image recognition techniques, can be utilized to assess fruit maturity. For instance, the YOLOC-tiny model is employed to determine citrus maturity, whereas VGGNet-16 is used to assess papaya ripeness (Ee Soong et al., 2024; Zuoliang et al., 2024). The development of lotus seeds begins 5 days after flowering and matures completely in about 30 days (Tu et al., 2020). However, no studies on this assessment of lotus seeds have been reported. Previous research has indicated that the texture of lotus seeds is primarily determined by the spatial and temporal distribution of cell wall polysaccharides, including hardness, starch, soluble sugars, pectin, cellulose, and hemicellulose (Mingjing et al., 2020). The ratio of amylose to amylopectin directly influences the “stickiness” of the seeds: a higher ratio tends to result in a harder texture, whereas a lower ratio can make them overly sticky; only a ratio close to 0.45–0.50 can achieve the desired mouthfeel (Chuanjie et al., 2023). The degradation and remodeling of pectin and cellulose directly alter the hardness and chewiness of the fruit (Fengjie et al., 2019). As fruit texture and nutrient components change during development and stabilize at maturity, they often require destructive sampling for detection, making them unsuitable as indicators for the batch detection of lotus seed ripeness.

In addition to textural changes, many fruits develop unique flavors during maturation, which are not present during their developmental process. The formation of flavor quality depends on the types and abundance of volatile organic compounds (VOCs), including esters, terpenes, alcohols, and aldehydes, which typically contribute to pleasant fragrances such as floral, fruity, and dairy scents (Yuanping et al., 2025). Fragrance characteristics are critical indicators of fruit maturation. Recent studies have revealed the fragrance characteristics of strawberries, peppers, kiwis, and grapes during maturation, which can serve as important criteria for determining their maturity (Wang et al., 2025; Yuanping et al., 2025; Yujie et al., 2025; Hang et al., 2026; Jiangfei et al., 2026). However, such research on lotus nuts is relatively rare.

Therefore, in this study, “JianXuan 30” was employed as the experimental material, and sequential sampling (5, 10, 15, 20, 25, and 30 days post-flowering) was performed to systematically integrate the phenotypic and agronomic traits, nutritional quality (soluble protein, soluble sugar, vitamin C, and total starch contents), cell wall components (pectin and cellulose), texture (hardness), and VOCs that are characteristic at the peak of maturity and are closely associated with differentially expressed genes. The aim is to elucidate the dynamic changes in nutrition, flavor, and texture during lotus seed development, and to establish associations between key VOCs, differentially expressed genes (DEGs), and maturity, thereby identifying molecular markers for precise harvesting. This comprehensive approach provides a theoretical foundation for the precise harvesting of lotus seeds.

## Results

### Investigation of the sensory quality of lotus seeds at six ripening stages

**Figure 1:**
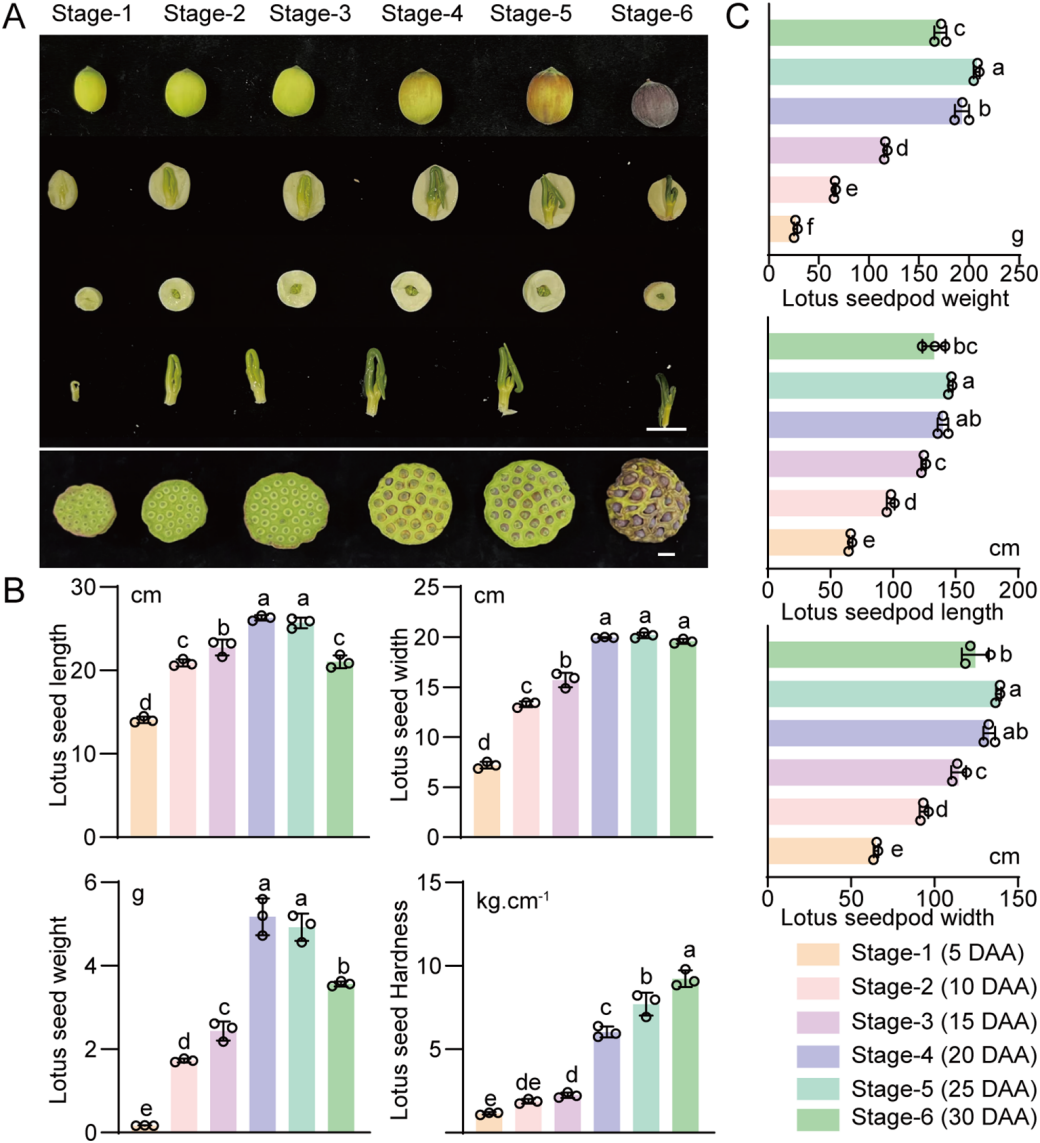
Changes in external traits during lotus seed and fruit development. **(A)** Morphological characteristics of “JianXuan 30” lotus pods and seeds. Bar=2 cm (top panel) and 5 cm (bottom panel). **(B)** Trends for lotus seed dimensions, weights, and hardness. **(C)** Trends for lotus seedpod dimensions and weights. The data presented are the means and standard errors of three replicates. Different lowercase letters indicate significant differences (p<0.05); uppercase letters indicate highly significant differences (*p* < 0.01).

Color is an important sensory quality indicator of the maturity of lotus seeds. We observed the color of lotus seeds at different ripening stages and found that the color of the lotus seeds changed from yellowish green (stages 1 to 3) to brownish green (stages 4 and 5) and then to dark purple (stage 6) (**Figure 1A**). Size and weight are also important sensory quality indicators for measuring the commercial value of lotus seeds and directly impact the purchase desire of consumers. Therefore, we measured lotus seed size parameters, including width and length. The width at stage 4 was significantly greater than that at the preceding stages, and no significant change was observed during lotus seed ripening; interestingly, the length of the lotus seeds first tended to increase at stages 4 and 5 but decreased at stage 6 (**Figure 1B**). Similarly, the weight tended to increase consistently from stage 1 to stage 4 but decreased at stage 6 as the lotus seeds ripened (**Figure 1C**). Hardness is an important sensory quality indicator of the texture and taste of lotus seeds. We measured lotus seed hardness and found that the values tended to increase consistently with increasing lotus seed growth and ripening. A hardness of 7.71 kg·cm^-1^ is the critical threshold of commercial hardness for lotus seeds at stage 5 suitable for drying, whereas the hardness of lotus seeds at stage 4 is more suitable for fresh consumption (**Figure 1D**) (Tu et al., 2020). Based on the results of the sensory quality assessment described above, we divided the six ripening stages into three phases: stage 1 to stage 3 were classified as the rapid enlargement phase, during which the yellowish green lotus seeds grew rapidly in size and weight and gradually took shape, and stages 4 and 5 were classified as the maturation phase, during which the brownish green lotus seeds reached their maximum size and weight and attained their optimal appearance and hardness.

Additionally, the size and appearance of lotus seed pods serve as direct visual indicators for identifying the optimal harvest period. We investigated the relationship between the maturity state of lotus seeds and the appearance of lotus seed pods by observing the lotus seed pod color and measuring their size and weight. Consistent with the changes observed in lotus seeds, lotus seed pods also presented a brownish-green color in stages 4 and 5, with their size and weight reaching their maximum values, indicating that these stages were the optimal time for harvest (**Figures 1F–H**).

### Assessment of the nutritional quality of lotus seeds at the six ripening stages

**Figure 2:**
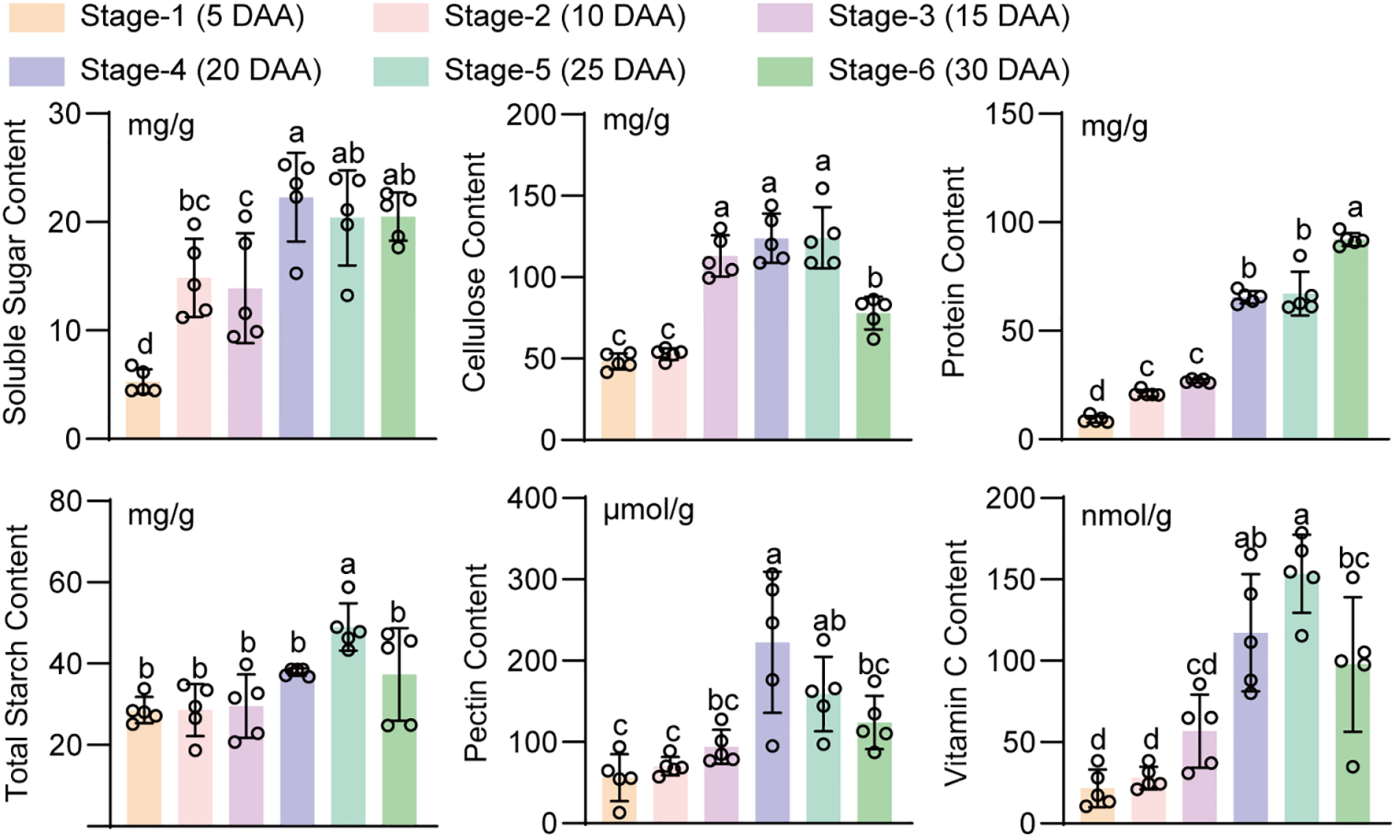
Changes in the contents of nutrients and cell wall components during lotus seed and fruit development. The data are presented as the means and standard errors of six replicates. Different lowercase letters above the bars indicate significant differences between means (*p* < 0.01), as determined using Fisher’s protected least significant difference (LSD) test.

Lotus seeds are rich in various nutrients, and their contents have important effects on their taste and nutritional quality (Tu et al., 2020). Therefore, we determined the contents of soluble sugars, starch, cell wall components, proteins, and vitamin C in lotus seeds during the six ripening stages (**Figure 2**). The content of soluble sugar, which is a major source of sweet taste, in lotus seeds initially tended to increase, peaking at stages 4 and 5 and then decreasing at stage 6, indicating that the sweetness was greatest in stages 4 and 5. A significant increase in starch accumulation was detected at stage 5, which may have improved the starchy and glutinous texture of the lotus seeds. However, in stage 6, the starch content decreased, which may have led to a softer texture of the lotus seeds and a decrease in quality. Protein and vitamin C are also important nutrients in lotus seeds. We found that the protein content in lotus seeds continued to increase, peaking at stage 6, whereas the vitamin C content first increased but then decreased, peaking at stage 4. These results indicate that although lotus seeds have a high soluble protein content in stage 6, stages 4–5 are considered the optimal harvest period because they not only maintain the best sweetness and good starchy and glutinous texture but are also rich in vitamin C. Moreover, the harvested lotus seeds at this time are also suitable for processing (Jia et al., 2026).

Cell wall components affect the hardness, morphology, and storability of lotus seeds. The contents of both cellulose and pectin among the cell wall components first increased but then decreased. The cellulose content reached its peak during stages 3–5, whereas the pectin content reached its peak during stages 4 and 5. These findings indicate that the increases in cellulose and pectin contents during stages 3–5 provides a solid cell wall structure for the fruit, which helps maintain the firmness and shape of the lotus seeds. The degradation of cellulose and pectin in stage 6 may lead to a decrease in lotus seed firmness and decreases in quality and storability. Therefore, for the best balance of taste, nutritional content, and storability, harvesting lotus seeds during stages 4 to 5 is recommended.

### Metabolomics analysis of the volatile compounds in lotus seeds

**Figure 3:**
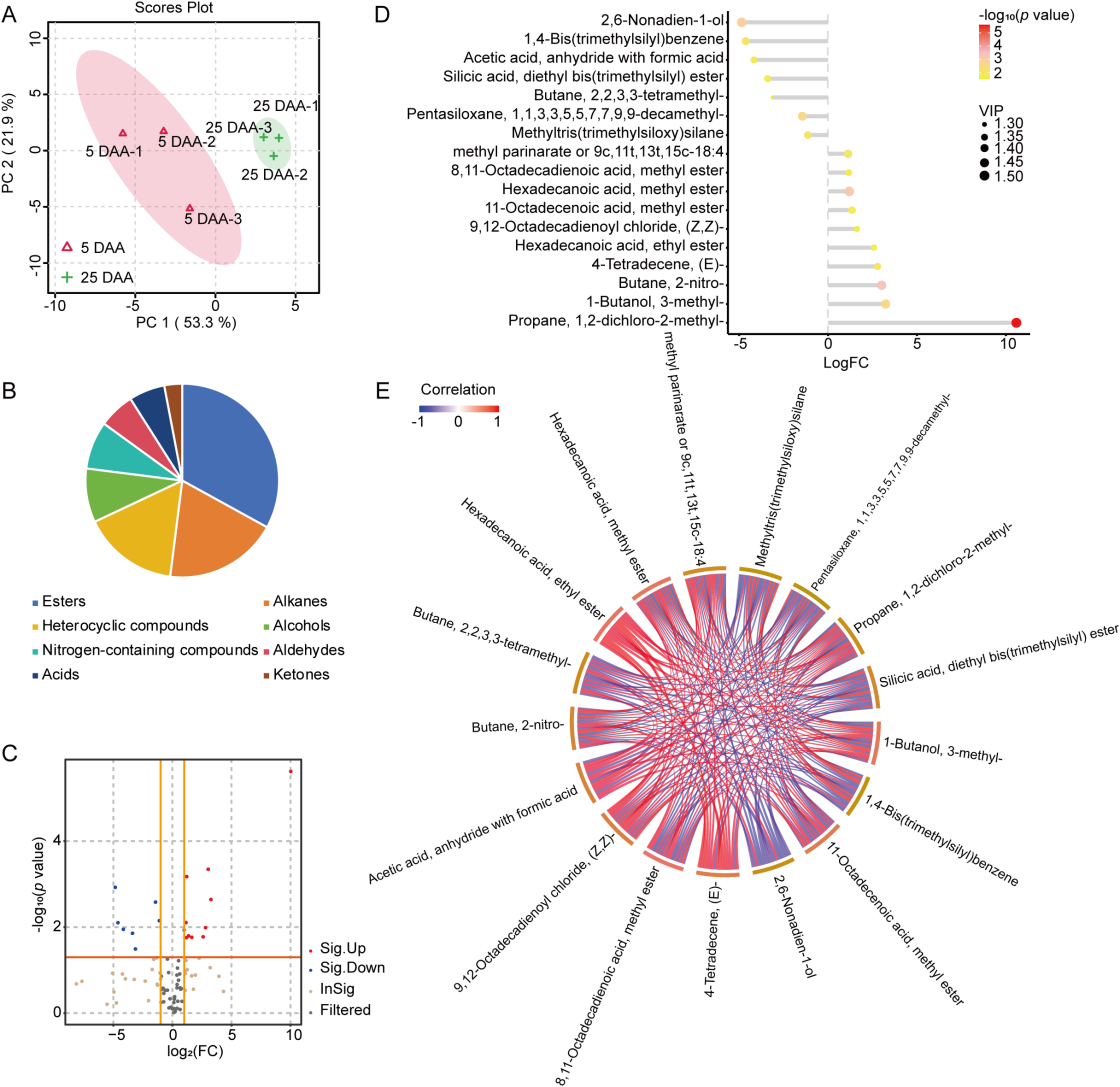
Analysis of volatile organic compounds (VOCs) in fragrant and nonfragrant lotus seeds. **(A)** Principal component analysis (PCA) based on HS-SPME-GC/MS. **(B)** Patterns of accumulation of different categories of VOCs. **(C)** Volcano plots displaying 14 significantly differentially abundant metabolites. **(D)** Matchstick diagrams illustrating the names of the differentially metabolized compounds, their *p* values, and their VIP values. **(E)** The strong correlation network of metabolites with correlation coefficients ≥0.85 and *p ≤* 0.05 is presented.

The fragrance of lotus seeds provides them with significant commercial value. We employed HS–SPME–GC–MS to analyze the volatile flavor components in nonaromatic 5 days after anthesis (DAA) lotus seeds and aromatic 25 DAA lotus seeds and to further elucidate the mechanism of flavor formation in lotus seeds. PCA (principal component analysis) results (**Figure 3A**) revealed that the three biological replicates of nonaromatic lotus (5 DAA) clustered together in the same category, while the three biological replicates of aromatic lotus (25 DAA) also clustered together, indicating good reproducibility.

A total of 82 volatile compounds, namely, esters (21 compounds, 33%), alkanes (12 compounds, 19%), heterocyclic compounds (10 compounds, 16%), alcohols (6 compounds, 9%), nitrogen-containing compounds (5 compounds, 8%), aldehydes (4 compounds, 6%), acids (4 compounds, 6%), and ketones (2 compounds, 3%), were detected in both scented and nonscented lotus seeds. Esters accounted for the highest proportion, followed by alkanes and heterocyclic compounds. These four categories constitute the primary components of the volatile compounds in lotus seeds (**Figure 3B**).

We compared the variability in the VOCs in samples 5 DAA and 25 DAA (**Figure 3C**), identifying 14 significantly altered VOCs (VIP > 1 and *p* < 0.05) that can be further categorized into aliphatic derivatives, aromatic derivatives, and compounds containing oxygen or nitrogen (**Figure 3D**, **Table 1**).

**Table 1:** Detailed information on the 14 significantly differentially abundant VOCs in lotus seeds.

	Names	R.T. (min)	CAS	Relative amount		Up/down
				A	B	
Fatty acid derivatives						
1	8,11-Octadecadienoic acid, methyl ester	30.056	56599-58-7	1.08	2.43	Up
2	Hexadecanoic acid, ethyl ester	28.689	628-97-7	0.13	0.79	Up
3	Hexadecanoic acid, methyl ester	27.773	112-39-0	10.38	23.76	Up
4	11-Octadecenoic acid, methyl ester	30.091	52380-33-3	0.75	1.92	Up
5	9,12-Octadecadienoyl chloride, (Z,Z)-	30.917	7459-33-8	0.02	0.09	Up
6	Methylparinarate (9c,11t,13t,15c-18:4)	30.743	1000336-46-6	0.13	0.29	Up
7	2,6-Nonadien-1-ol	14.287	7786-44-9	0.05	0	Down
8	4-Tetradecene, (E)-	19.161	41446-78-0	0.01	0.08	Up
9	Butane, 2,2,3,3-tetramethyl-	1.478	594-82-1	37.1	4.15	Down
Aromatic derivatives						
10	1,4-Bis(trimethylsilyl)benzene	12.64	13183-70-5	0.09	0	Down
Nitrogen and oxygen heterocyclic compounds						
11	1-Butanol, 3-methyl-	3.221	123-51-3	0.08	0.77	Up
12	Acetic acid, anhydride with formic acid	2.669	2258-42-6	0.43	0.02	Down
13	Butane, 2-nitro-	3.22	600-24-8	0.05	0.4	Up
14	Propane, 1,2-dichloro-2-methyl-	2.837	594-37-6	0	13.29	Up

We noted that the abundance of seven aliphatic derivatives in 25 DAA was significantly higher than that in 5 DAA, suggesting that the difference in the accumulation of volatile aliphatic derivatives may be closely related to the formation of the lotus fragrance. We further screened the strong correlation network of all the metabolites with correlations ≥ 0.85 and *p* ≤ 0.05 as the threshold and identified four key metabolite nodes with the highest connectivity and central position in the overall network, using the “frequency of occurrence” as a topological indicator. Among these metabolites, the frequency of occurrence of butane, 2-nitro- (9), hexadecanoic acid, methylester (9), 1-butanol, 3-methyl- (9) and methylparinarate (8) was the highest among all the VOCs, suggesting that these VOCs are the key metabolite nodes for the production of lotus fragrance (**Figure 3E**). Chemically, the highly positive correlation of the cluster between fatty acid methyl esters (hexadecanoic acid methyl esters and methylparinarate) with r > 0.9 indicates that they are driven by the same synthetic or β-oxidation pathway. The synchronous changes in the alcohol (1-butanol, 3-methyl-) and the fatty acid clusters suggest that alcohols may be a downstream product of lipid degradation. In summary, hexadecanoic acid methylester, 1-butanol, 3-methyl, and methylparinarate constitute the core triangle of the differentially abundant metabolic network.

Based on the results of the correlation analysis of the aforementioned differentially abundant metabolites, we further mapped the key metabolic nodes with high connectivity and strong correlations onto pathways in the KEGG database. These metabolites were enriched primarily in core lipid metabolic pathways such as fatty acid biosynthesis (ko00061), unsaturated fatty acid biosynthesis (ko01040), fatty acid metabolism (ko01212), and butyrate metabolism (ko00650) (**Table 2**). These key VOCs are involved mainly in biological processes such as lipid synthesis, regulation of unsaturated fatty acids, and short-chain alcohol metabolism, suggesting that the reprogramming of lipid metabolism may be critical for aroma production. Hexadecanoic acid methyl ester, a representative saturated fatty acid methyl ester, is a key intermediate in the fatty acid biosynthesis pathway, and its high connectivity indicates its potential role as a hub involved in lipid synthesis and energy storage. Methylparinarate, an enoic acid, is enriched in the unsaturated fatty acid biosynthesis pathway and may participate in the regulation of membrane lipid structure and signal transduction processes. 11-Octadecenoic acid methyl ester, which is enriched in the fatty acid metabolism pathway, is potentially involved in the β-oxidation of fatty acids or membrane lipid remodeling processes. Moreover, 1-butanol, 3-methyl-, a branched-chain alcohol metabolite, is enriched in the butyrate metabolism pathway, indicating that its possible origin is the degradation of fatty acids or amino acids.

**Table 2:** Key metabolites detected in lotus seeds that were mapped to KEGG pathways.

Path name	KEGG ID	Enriched metabolites	Research significance
Fatty acid biosynthesis	ko00061	Hexadecanoic acid methyl ester	Core pathways of lipid synthesis
Biosynthesis of unsaturated fatty acids	ko01040	Methylparinarate	Membrane lipid regulation and signal transduction
Fatty acid metabolism	ko01212	11-Octadecenoic acid methyl ester	Energy metabolism, membrane composition
Butanoate metabolism	ko00650	1-Butanol, 3-methyl-	Short-chain alcohol metabolism, lipid degradation

In summary, the differences in the contents of lipid substances represented by hexadecanoic acid methyl ester significantly affects the formation of lotus seed flavor.

### RNA-seq analysis of lotus seeds at different stages of ripening

The alterations in metabolic compounds are closely associated with the differential expression of related genes. As a method to further elucidate the reasons for the discrepancies in the VOC contents, we utilized RNA-seq to investigate the changes in gene expression between 5 DAA and at 25 DAA. Using the Illumina NovaSeq 6000 platform, paired-end sequencing (2×150 bp) was performed on six cDNA libraries derived from lotus seeds. After the adapter sequences, low-quality reads, and redundant sequences were removed, a total of 38.89 Gb of clean data were obtained; the amount of clean data per sample ranged from 6.41 to 7.65 Gb, with an average of 6.48 Gb. The Q30 value was greater than 97.78% for all the samples, with an average Q30 of 97.96%. The clean reads from each sample were aligned to the designated reference genome, with alignment rates ranging from 93.52% to 95.48% (**Supplementary Table S1**). The data quality was excellent and met the requirements for subsequent transcriptomic analyses.

**Figure 4:**
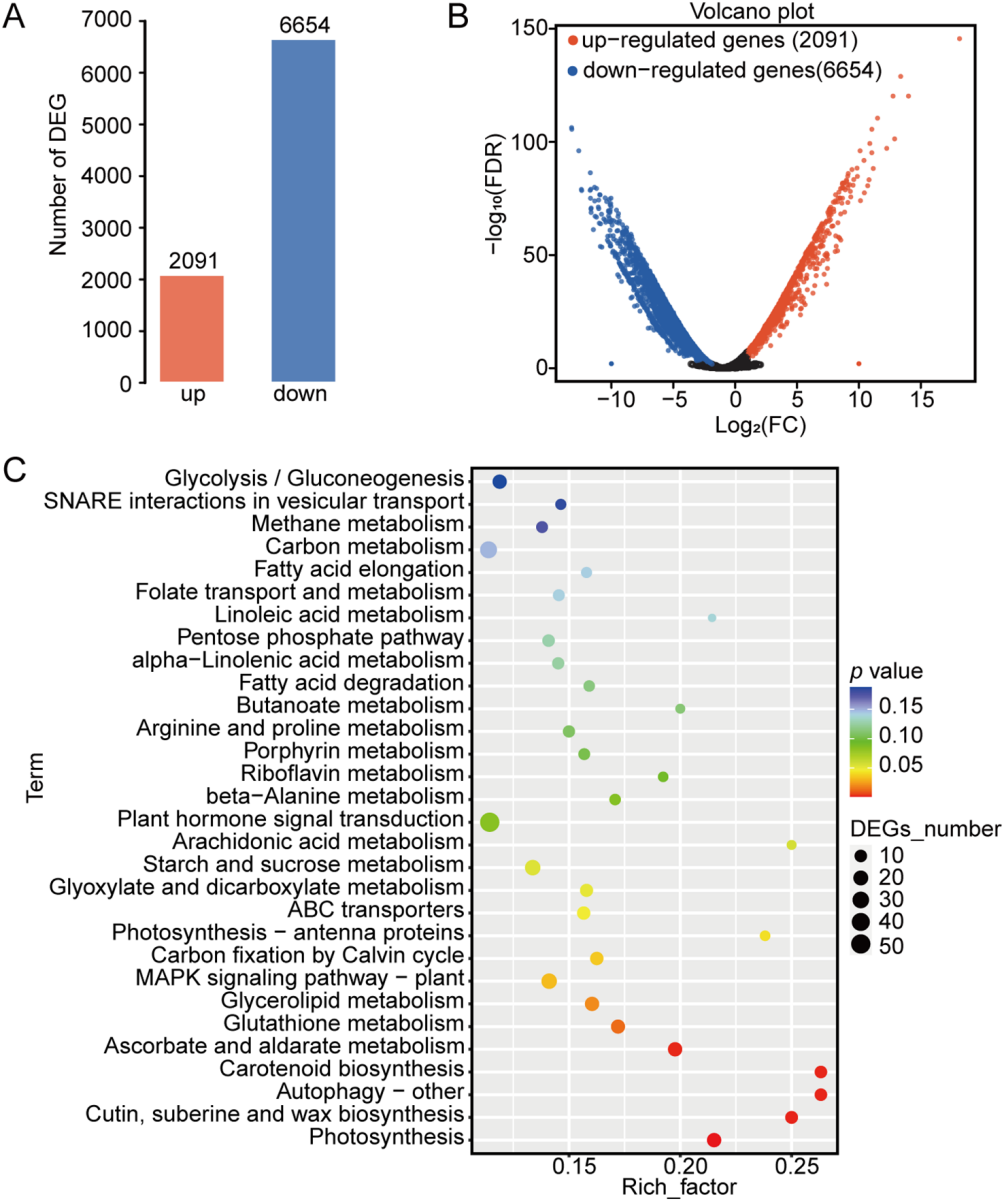
Analysis of differentially expressed genes (DEGs) in nonfragrant and fragrant lotus seeds. **(A)** Numbers of upregulated and downregulated DEGs in nonfragrant compared with fragrant lotus seeds. **(B)** Volcano plot of upregulated and downregulated DEGs in nonfragrant compared with fragrant lotus seeds. **(C)** KEGG pathways enriched in DEGs that were upregulated in fragrant **(B)** and nonfragrant **(A)** lotus seeds.

A total of 8,745 differentially expressed genes (DEGs) were identified between the lotus seeds from samples 5 DAA and 25 DAA (**Figure 4A**). Among these genes, 2,091 genes were upregulated and 6,654 genes were downregulated in the lotus seeds of 25 DAA compared with those of 5 DAA (**Figure 4B**). We primarily focused on the 2,091 upregulated DEGs. Consistent with the results of the analysis of the differentially abundant VOCs, the results of the KEGG pathway enrichment analysis of the upregulated differentially expressed genes revealed that they were significantly enriched in the fatty acid metabolism pathway (**Figure 4C**).

**Figure 5:**
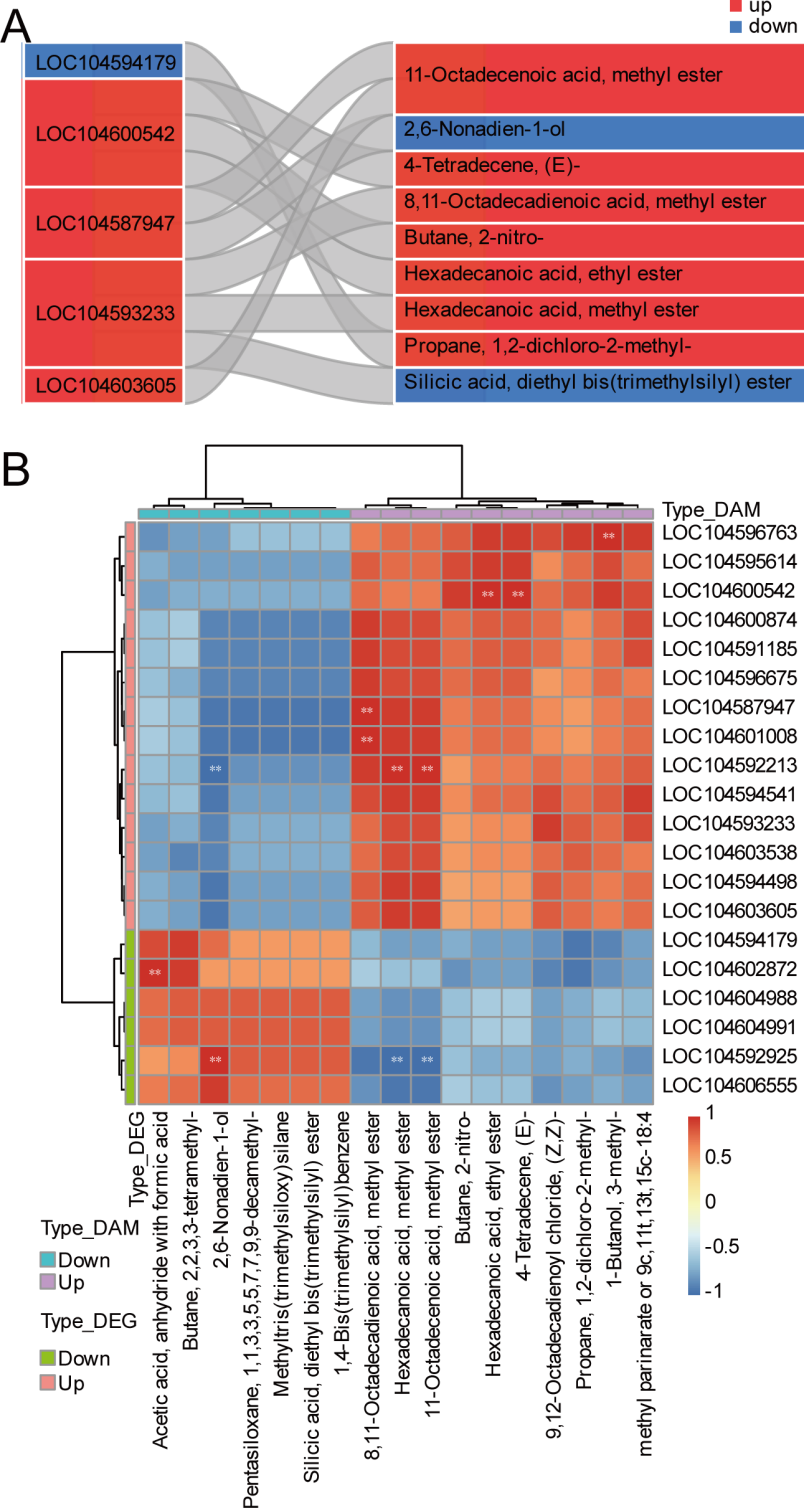
O2PLS analysis of differentially expressed genes and differentially abundant metabolites in nonfragrant and fragrant lotus seeds. **(A)** Sankey diagram of the top 20 differentially expressed genes and metabolites, with the left side representing differentially expressed genes and the right side representing differentially expressed metabolites. **(B)** Correlation heatmap of differentially expressed genes and differentially abundant metabolites. The horizontal and vertical coordinates represent the key genes and metabolites used for the analysis, respectively. The color of the blocks in the heatmap represents the correlation coefficient obtained using Pearson’s correlation analysis. Red represents a positive correlation, blue represents a negative correlation, and “*” indicates that the differentially expressed genes and differentially abundant metabolites are significantly correlated (*p* value < 0.05). “**” indicates that the differentially expressed genes and differentially abundant metabolites are extremely significantly correlated (*p* value < 0.01).

We correlated the metabolomic and transcriptomic results to further associate VOCs with differentially expressed genes and identify the DEGs responsible for the variations in the contents of key VOCs. Through the O2PLS analysis (Two-Way Orthogonal Partial Least Squares), we identified differentially expressed genes and metabolites with strong correlations in both omics analyses. We found that *LOX* (LOC104600542), *ADH* (LOC104587947), and ALDH (LOC104593233) were strongly correlated with the levels of butane, 2-nitro-, hexadecanoic acid, methylester, 1-butanol, 3-methyl-, and methylparinarate (**Figure 5A**). The expression levels of these genes and the contents of the metabolites were significantly higher in the lotus seeds from 25 DAA than those in 5 DAA (**Figure 5B**).

**Figure 6:**
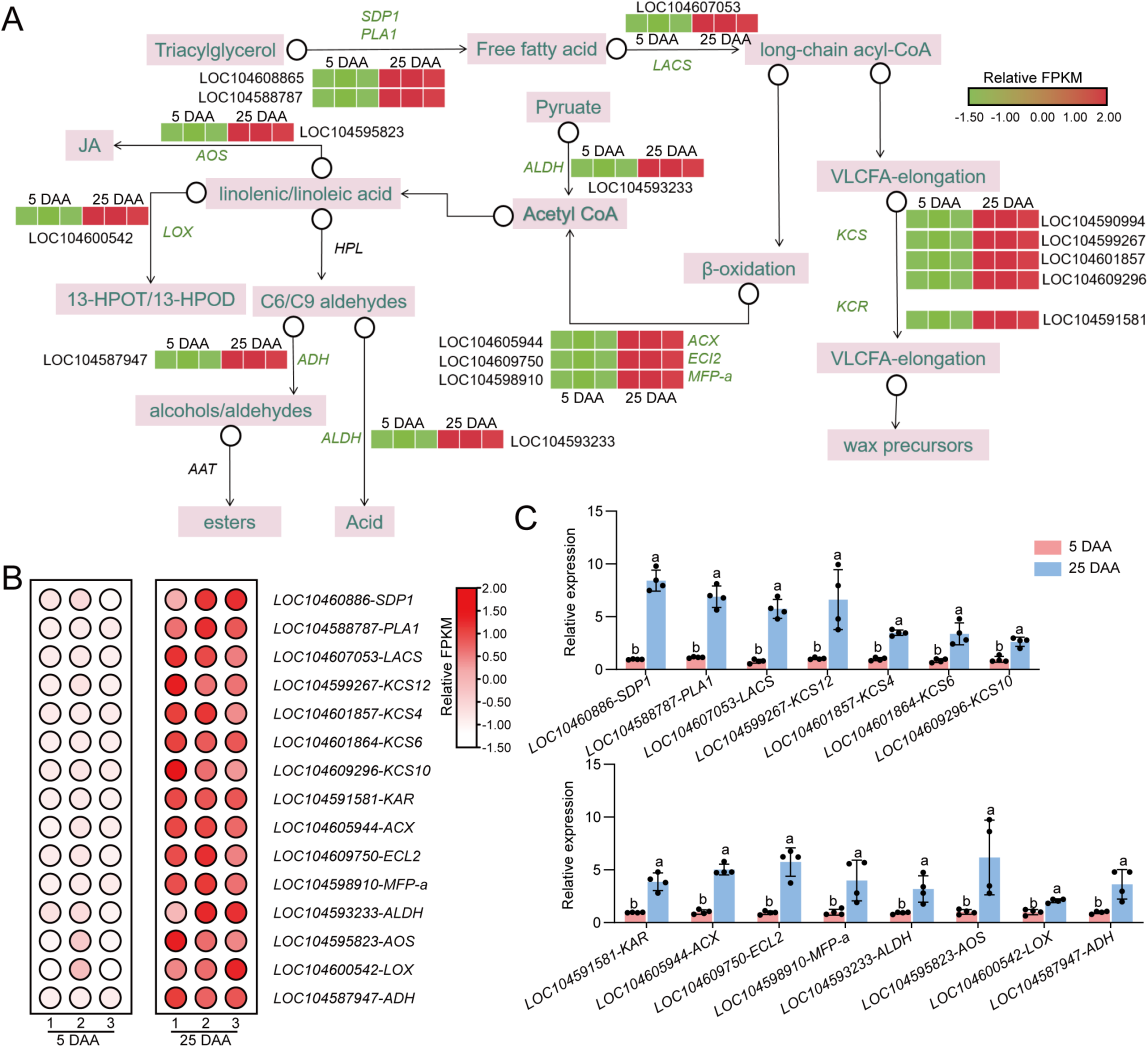
Fatty acid synthesis pathway in lotus seeds. **(A)** Differentially expressed genes in the fatty acid synthesis pathway. **(B)** Heatmap showing the FPKM values of differentially expressed genes involved in fatty acid metabolism. **(C)** qRT–PCR validation of the transcriptomic results. The data presented are the means and standard errors of four replicates. Different lowercase letters above the bars indicate significant differences between means (*p* < 0.01), as determined using t tests. FPKM, Fragments Per Kilobase of exon model per Million mapped fragments.

We further examined the differentially expressed genes that were enriched in the fatty acid metabolic signaling pathway (**Figure 6A**). We observed that other genes closely associated with the fatty acid metabolic signaling pathway were also upregulated during 25 DAA, leading to significant changes in the levels of lipid substances during this period (**Figure 6B**). We further validated the transcriptomic results using quantitative real-time PCR. The results indicated that the expression levels of *LOX*, *ADH*, and *ALDH* were significantly higher during 25 DAA than during period A, consistent with the transcriptomic findings (**Figure 6C**). By examining the expression levels of these genes across six stages, it was discovered that they begin to be transcriptionally activated at 20 DAA, which is highly correlated with the ripening of lotus seeds (**Supplementary Figure S1**).

**Figure 7:**
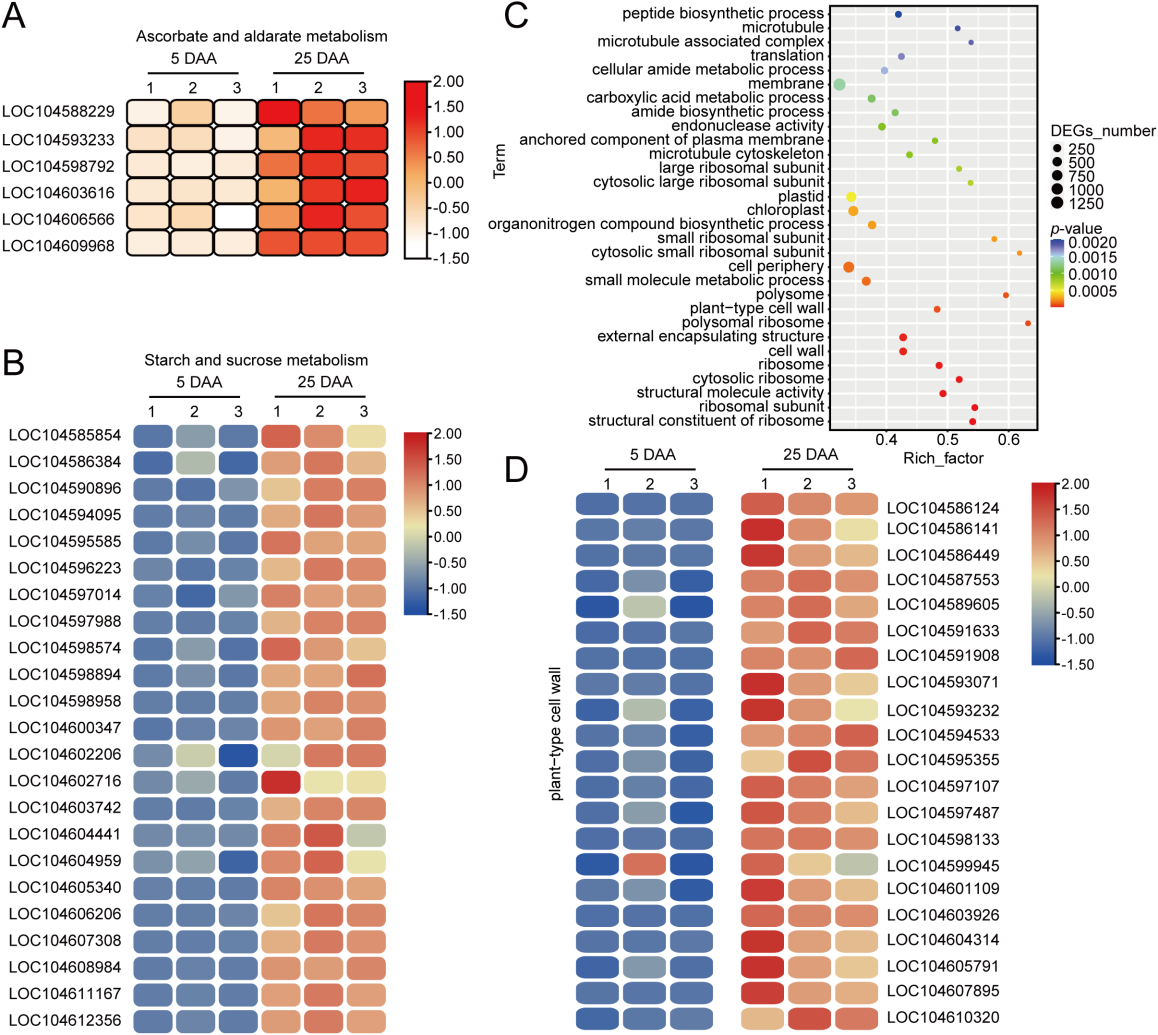
Fatty acid synthesis pathway in lotus seeds. **(A)** Heatmap showing the FPKM values of differentially expressed genes involved in ascorbate and aldarate metabolism. **(B)** Heatmap showing the FPKM values of differentially expressed genes involved in starch and sucrose metabolism. **(C)** GO enrichment analysis of DEGs that were upregulated in fragrant **(B)** and nonfragrant **(A)** lotus seeds. **(D)** Heatmap showing the FPKM values of differentially expressed genes enriched in components of the cell wall.

In addition to changes in flavor, some important lotus seed qualities also formed simultaneously during 25 DAA. According to the results of the KEGG enrichment analysis, we noted that pathways related to “ascorbate and aldarate metabolism” were significantly enriched, which led to an increase in the vitamin C content (**Figure 7A**; **Supplementary Table S2**), consistent with our previous findings. Several upregulated genes were closely related to starch and sugar synthesis, and their transcriptional activation leads to starch accumulation. The changes in the quality of lotus fruits at different maturation stages observed previously may be closely related to the expression of these genes (**Figure 7B**). We also conducted a GO enrichment analysis on the DEGs and found that functional modules related to cell wall synthesis were significantly enriched, which may be crucial factors leading to the increase in the expression of cell wall-related metabolites such as cellulose and pectin, thereby promoting the increase in lotus seed hardness (**Figures 7C, D**; **Supplementary Table S3**).

## Discussion

High-quality lotus seeds are not only a delightful food but also a category of medicinal herbs. Unlike common fruits, lotus seeds are encapsulated within the lotus pod and are released only after the seeds have fully matured, a process that often results in the seeds losing their culinary value. Understanding the optimal harvesting period and the changes in volatile compounds in lotus seeds at different developmental stages is crucial for understanding their quality and for utilizing an electronic nose to assist in harvesting. Our findings indicate that the most suitable time for harvesting lotus seeds is between 20 DAA and 25 DAA, during which the quality of the seeds is fully established. Furthermore, by combining transcriptomics and metabolomics, we elucidated the characteristic VOCs present in fully mature lotus seeds and identified differentially expressed genes that are closely associated with these VOCs, laying the foundation for a molecular description of lotus seed maturation and quality formation.

### The morphological and nutritional characteristics of mature lotus seeds

The maturation of plant fruits is accompanied by changes in appearance and texture, as well as the continuous accumulation of nutrients and flavor compounds until full development (Graham B et al., 2013). However, once the fruit reaches full development, its quality gradually deteriorates. Therefore, an accurate judgment of the harvest time is crucial in agricultural production.

We observed that during the 5 to 25 days after flowering, the size of the fruit and the lotus pod of “JianXuan 30” lotus seeds gradually increased, characterized by sustained growth in both the longitudinal and transverse diameters. The longitudinal and transverse diameters of the lotus seed fruits reached their maximum values between 20 and 25 days postflowering, at which point the fruits were fully expanded and morphologically stable. These findings are consistent with those of previous reports on other lotus seed varieties, suggesting that the period of lotus seed development is approximately 30 days (Zhongyuan et al., 2019). Furthermore, the rate of fruit expansion in lotus seeds is inconsistent throughout their entire growth period. After 5 DDA, the longitudinal and transverse diameters as well as the weight of the lotus seeds increased rapidly, and the fruit shape gradually formed. However, after 15 DDA, the morphological changes in the lotus seeds slowed, although their weight continued to increase gradually.

Lotus seeds are rich in a variety of nutrients, including carbohydrates, proteins, and vitamin C (Bo et al., 2009). Alterations in the cell wall structure can lead to changes in the texture of lotus seeds. Through the detection of nutritional components and cell wall components in lotus seeds, we found that the contents of certain nutrients, such as soluble sugars, proteins, starch, and vitamin C, increased significantly during the fruit expansion period, but rapidly increased after 15 DDA, with this process lasting for 10 days. These findings are consistent with previous observations of lotus seeds by other researchers. Sun et al. divided the development cycle of lotus seeds into four stages: the organ formation stage (1 to 3 days after pollination), the cell expansion stage (4 to 9 days), the nutrient accumulation stage (10 to 25 days), and the dehydration maturation stage (26 to 30 days). Compared with fruits that can be directly observed, lotus seeds are hidden within the lotus pod, and delayed harvesting can lead to a substantial degradation of their quality (Zhixia et al., 2024; Jia et al., 2026). Therefore, accurately determining whether lotus seeds are ripe is highly important. The results of this study indicate that judging the ripeness of lotus seeds solely based on appearance is challenging. If harvested prematurely, the accumulation of nutrients in lotus seeds may be insufficient. Additionally, other studies have shown that unripe lotus seeds exhibit significantly reduced processing characteristics and starch quality.

In summary, we have described the morphological development and accumulation of nutritional substances during the growth and development of lotus seeds, revealing the asynchronicity of the development of the external morphology and accumulation of nutritional substances during the process of seed development.

### Lotus seeds release aromatic substances dominated by fatty acids when they reach maturity

Given that the maturity of lotus seeds cannot be directly determined by their appearance appropriate characteristic indicators must be used to assess their maturity. This approach will aid in the harvesting and assessment of the quality of lotus seeds. Current research on the characteristics of mature lotus seeds focuses mainly on morphology, nutritional quality, and processing quality (Zhixia et al., 2024). The detection of these indicators often results in damage to the samples, which limits their practical application.

Apart from changes in appearance and nutritional components, the physiological characteristics of fruits change as they reach maturity, leading to significant differences in the volatile compounds they emit. For example, during the ripening of kiwifruit, the dominant VOCs gradually shift from aldehydes to terpenoids (such as β-ionone and β-damascenone) and esters (Qingchao et al., 2025; Lijuan et al., 2026). During the ripening process of Euryale ferox, the types and contents of flavonoids and phenolic acids will increase (Liu et al., 2023). Similarly, fully mature lotus seeds also have a subtle aroma (Shuang et al., 2025). Using HS–SPME–GC–MS, we found that compared with those of unripe lotus seeds, 14 significantly differentially abundant VOCs were emitted by mature lotus seeds, with esters being the main component. Ester compounds typically possess pleasant odors such as fruity, floral, and sweet fragrances, which are the primary sources of fruit aroma (Paul K et al., 2015). During the ripening of strawberries, the dominant VOCs transition from aldehydes to esters and terpenoids (Yujie et al., 2025). In ripe yellow peaches, increased contents of esters such as ethyl acetate and γ-decalactone contribute to their rich fruit aroma (Honggang et al., 2023). Our results indicate that butane, 2-nitro-, hexadecanoic acid, methylester, 1-butanol, 3-methyl-, and methylparinarate are the main VOCs emitted by mature lotus seeds. 3-Methylbutanol, a VOC also produced by tomatoes, apples, and grapes as they ripen, is formed from leucine through Strecker degradation and has been shown to be associated with resistance to gray mould in grapes (Sonia A et al., 2013; Jianing et al., 2025; Shunjie et al., 2025). Methyl palmitate, another important flavor compound, has also been detected as a significantly differentially abundant VOC in the fermentation of durian, tea, and chili (Jian et al., 2024; Lei et al., 2025; Yunwei et al., 2026). These compounds may serve as characteristic substances for the nondestructive detection of the maturity of lotus seeds in the future.

### LOX, ADH, and ALDH are closely related to the formation of VOCs in lotus seeds

During fruit ripening, numerous transcriptional reprogramming events occur, leading to significant changes in the physiological and biochemical activities of the fruit. The biochemical synthesis of VOCs in plant fruits is closely associated with transcriptional regulation (Jesús et al., 2020). During pepper ripening, VOC synthesis is closely related to increased expression levels of genes such as CaLOX and CaADHL (Yinhui et al., 2023). In this study, we utilized a combination of transcriptomic and metabolomic analyses to elucidate the differentially expressed genes closely related to the formation of VOCs in lotus seeds. We identified 2091 upregulated differentially expressed genes, and the KEGG enrichment analysis indicated that these genes are closely related to fatty acid metabolism. Through an O2PLS analysis, we found that LOX, ADH, and ALDH are closely related to the synthesis of butane, 2-nitro-, hexadecanoic acid, methylester, 1-butanol, 3-methyl-, and methylparinarate. Lipoxygenase (LOX) is the rate-limiting enzyme involved in the biosynthesis of fatty acid-derived VOCs, and its expression is closely related to the release of fatty acid-derived VOCs in horticultural crops (Md Sanaullah and Jun'ichi, 2021). The specific fatty acid metabolism pathway mediated by LOX/ADH/ALDH centers around the initiation of polyunsaturated fatty acid (PUFA) oxidation by LOX, followed by the generation of aldehydes through hydroperoxide lyase (HPL) cleavage. These aldehydes are then sequentially reduced/oxidized by ADH and ALDH, ultimately leading to the production of volatile alcohols, acids, and signaling molecules. This pathway is crucial for the formation of plant aroma. The expression of LcLOX7 in litchi endows its fruit with a fresh aroma (Zhuoyi et al.). The hypoxic stress-related gene MdASG1 mediates the accumulation of volatile aroma compounds in apples by activating MdLOX1a (Jing et al., 2024). The upregulated expression of the alcohol dehydrogenase (ADH) gene can significantly increase the conversion of aldehydes to alcohols. The main aromatic components in Osmanthus are monoterpenes, and the enzymatic oxidation of monoterpene alcohols is significantly correlated with the ADH content. The expression of the ADH gene in Osmanthus varies across seasons, thus endowing Osmanthus with different aromas during different seasons (Jiani et al., 2023). The aldehyde dehydrogenase (ALDH) family, which includes NAD+- or NADP+-dependent enzymes, plays a crucial role in reducing the toxicity of aldehydes by converting them into their corresponding carboxylic acids. It functions in plant stress responses, and AaALDH1 is involved in artemisinin synthesis mediated by ABA and JA (Zhiying et al., 2023). These three genes can serve as important marker genes for the maturation of lotus seeds and have potential application value. In the future, their expression levels could be measured as supporting indicators for evaluating the aroma of fresh lotus seeds.

In addition to the genes closely related to VOCs, we utilized KEGG and GO enrichment analyses to identify differentially expressed genes associated with vitamin C biosynthesis and the biosynthesis of cellulose and pectin. Vitamin C is a crucial nutritional indicator in lotus seeds, and the KEGG enrichment analysis revealed that the expression levels of six genes involved in vitamin C biosynthesis were significantly increased during the maturation phase of lotus seeds. In kiwifruits, prickly pears, and other fruits, a substantial number of genes related to vitamin C biosynthesis are significantly activated during maturation, contributing to the high vitamin C content in ripe fruits (Ichiro et al., 2004; El Hassania et al., 2024). Cellulose and pectin are closely associated with the texture of fruit tissues, and the GO enrichment analysis revealed that the expression of genes involved in cellulose and pectin biosynthesis was markedly increased during the maturation of lotus seeds, indicating an increase in the hardness of the seeds at this stage (Min et al., 2009). In the future, by comparing the differences in the expression of these genes across different lotus seeds varieties and correlating them with the texture of the seeds, it may be possible to detect and predict the quality of lotus seeds at the molecular level.

In summary, we elucidated the changes in fruits during the development of lotus seeds from multiple dimensions, including morphology, the accumulation of nutritional substances, volatile metabolites, and differential gene expression. This study provides potential metabolite indicators and marker genes for the timely harvest of lotus seeds.

## Materials and methods

### Plant materials and growth conditions

Fresh samples of “JianXuan 30” lotus seeds were provided by the Jianning County Lotus Seed Research Institute. “JianXuan 30” is the main lotus seed variety cultivated locally in Jianning, with high yield and excellent flavor. Seed lotus should be planted in spring when the temperature is steadily above 15°C, at a density of 2.5–3.0 m row spacing and 1.2–1.5 m plant spacing. Water depth is managed as “shallow–deep–shallow” to promote germination, growth and seed maturation. Fertilization follows the principle of “stable early, sufficient middle, controlled late”, with split applications to ensure nutrient supply during flowering and seed setting. On March 29, 2024, the samples were planted at the institute’s new lotus variety breeding base (116°45′ E, 26°50′ N). Field management during the growth period followed standard cultivation practices. The day of full lotus flower bloom was recorded as Day 1 of the seed growth cycle (post flowering), after which the fruit development days were tracked. Fruits were harvested at six consecutive sampling points—5, 10, 15, 20, 25, and 30 DAA—with deformed fruits removed. Fruits with a consistent size and color were selected for the experiment, with three biological replicates established at each sampling point. Cross-sections and longitudinal sections of peeled or whole lotus seeds were photographed in ascending order of maturity. Freshly harvested lotus seeds were transported to the laboratory within two hours. The cotyledons and seeds were separated, and the lotus seed pulp was freeze-dried, ground into powder, and stored for later use.

### Measurement indicators and methods

#### Measurement of lotus seed phenotypic and agronomic traits

Using a GY-4 digital fruit hardness tester with a 5 mm diameter cylindrical probe, the hardness was measured at three points (top, middle, and bottom) of each fruit by pressing to a depth of 5 mm, and the average values were recorded. The fruit cross-sections (at the point of maximum transverse diameter) and longitudinal sections were photographed, and then the transverse and longitudinal diameters of the fruits were measured.

### Nutritional analysis of lotus seeds

A soluble protein assay kit (BC3185, Solarbio, Beijing), a soluble sugar assay kit (BC0035, Solarbio, Beijing), a vitamin C assay kit (BC1230, Solarbio, Beijing), a pectin assay kit (BC1405, Solarbio, Beijing), a cellulose assay kit (BC2545, Solarbio, Beijing), and an amylose/beta-starch/total starch content detection kit (BC6100, Solarbio, Beijing) were used in conjunction with a multifunctional microplate reader (TECAN/SPARK) according to the specific wavelength requirements for each indicator in the kit (Solarbio). The specific operational steps are described below. The standard solutions and sample solutions provided with the kits were prepared. The standard solution concentrations were 0.1, 0.2, 0.4, 0.8, and 1.6 mg/mL. The sample solution was prepared by dissolving lotus seed pulp powder in deionized water, followed by centrifugation (10,000 rpm, 10 min) to obtain the supernatant. The wavelength of the microplate reader was set according to the instructions provided with the kit for measurement. For example, the wavelength for measuring the soluble protein content is 595 nm, and it is 510 nm for measuring the soluble sugar content. The absorbance values were recorded, and the content of each nutrient in the sample was calculated using a standard curve. When preparing the standard curve, standard solutions were added to the reaction reagents provided in the kit, the absorbance values were measured, and the standard curve was plotted with the standard solution concentration on the x-axis and the absorbance values on the y-axis.

### Analysis of volatile components in lotus seeds

After removing the cotyledons from lotus seed fruits, the endosperm within the cotyledon cavity was immediately cryopreserved in liquid nitrogen and stored at -80 °C for later use. Volatile components were determined using headspace solid-phase microextraction (HS-SPME) coupled with gas chromatography–mass spectrometry (GC–MS). The procedure is described below. Approximately 500 mg of lotus seed fruit sample was weighed into a 20 mL headspace vial. A saturated NaCl solution was added to increase the efficiency of the release of volatile components, and 10 μL of deuterated toluene (toluene-d8) internal standard solution (50 μg/mL) was also added for the quantitative analysis. The vial was sealed and equilibrated in a 60 °C water bath for 10 min to allow the complete release of volatile components into the headspace. A 65 μm PDMS/DVB SPME head was selected because of its excellent adsorption properties toward various volatile organic compounds. The extraction temperature was set at 60 °C for 50 min to ensure the complete adsorption of volatile components. Following extraction, the SPME head was promptly inserted into the GC–MS inlet port. Desorption was performed at 250 °C for 5 min to release the adsorbed volatile components. The GC–MS analysis conditions were as follows: an HP-5MS capillary column (30 m × 0.25 mm × 0.25 μm) was used, which was suitable for separating various volatile organic compounds; high-purity helium was used as the carrier gas at a flow rate of 1.0 mL/min; and the temperature program consisted of an initial temperature of 40 °C held for 2 min, followed by a ramp to 250 °C at 5 °C/min, and a hold for 10 min. This program effectively separated volatile components with different boiling points. The injection port temperature was set to 250 °C with splitless injection. The ion source temperature of the mass spectrometer detector was 230 °C, the interface temperature was 280 °C, and the electron impact energy was 70 eV. Finally, the GC–MS results were compared with the NIST standard spectral library. The identification criteria required a similarity index (SI) and reverse similarity index (RSI) ≥8800 to determine volatile component types. The relative content of each component was determined using peak area normalization. For specific components, a quantitative analysis was performed using the internal standard method by calculating the ratio of peak areas between each volatile compound and the internal standard.

### RNA extraction and RT–qPCR analysis

The changes in the expression of key genes involved in three major pathways, starch synthesis, pectin/xylan metabolism, and fatty acid–volatile compound synthesis, during lotus seed development was validated using real-time quantitative PCR (RT–qPCR). Total RNA was extracted from lotus seeds at 5 and 25 days after anthesis (DAA) using TRIzol reagent (Invitrogen) according to the manufacturer’s protocol. Total RNA was extracted from fresh lotus seed tissues using the Trizol method. Fresh tissues were frozen in liquid nitrogen, ground into powder, and 0.1–0.2 g powder was mixed with 1 ml Trizol reagent. After incubation at room temperature for 10 min, 0.2 ml chloroform was added. Following centrifugation at 12000 g for 15 min at 4 °C, the upper aqueous phase was collected and mixed with 0.5–1 ml isopropanol. The RNA pellet was obtained after centrifugation at 12000 g for 10 min at 4 °C, then washed twice with 75% ethanol and air-dried. The pellet was resuspended in 15–30 μl RNase−free water.

The integrity and purity of the extracted RNA were assessed by electrophoresis on a 1% agarose gel, and the concentration was determined using a NanoDrop 2000 spectrophotometer (Thermo Fisher Scientific). One microgram of total RNA was reverse transcribed into cDNA using the PrimeScript™ RT Reagent Kit (TaKaRa) under the following conditions: 37 °C for 15 min and 85 °C for 5 s, followed by cooling to 4 °C. The resulting cDNA was diluted 10-fold for the qPCR analysis. Specific primers were designed using Primer Premier 6.0 software based on the sequences of known genes (the primer sequences are listed in **Supplementary Table S4**). All primers were synthesized by Shanghai Sangon Biotech Co. Ltd. qPCRs were performed using SYBR^®^ Premix Ex Taq™ II (TaKaRa). The reaction system was 20 μL and consisted of 10 μL of 2× SYBR Premix Ex Taq II, 0.4 μL of 50× ROX reference dye, 0.8 μL of forwards and reverse primers (10 μM), 2 μL of cDNA template, and 6 μL of ddH_2_O. The reaction conditions were 95 °C for 30 s, followed by 40 cycles of 95 °C for 5 s and 60 °C for 30 s. Each sample included three biological replicates and three technical replicates. RT–qPCR was performed using an ABI 7500 real-time fluorescent quantitative PCR instrument (Applied Biosystems), and the data were analyzed using ABI 7500 Software v2.0.6. Relative gene expression was calculated using the 2^-ΔΔCt^ method and normalized to the expression of the lotus housekeeping gene ACTIN2 (Xiaohan et al., 2022) (primer sequences: F—5’-GCGTTCTGCCGTCTTCTAAA-3’, R—5’-CCCTCTTGGATTGTGCCTC-3’). All the qPCR data are presented as the means and were analyzed using one-way analysis of variance (ANOVA) with SPSS 26.0 software to assess significant differences in gene expression.

### Transcriptome sequencing and data analysis

Transcriptome sequencing and basic data analysis were conducted by Ling’en Biotechnology Co., Ltd. (http://www.biozeron.com/). Total RNA was extracted using TRIzol reagent (Invitrogen). RNA integrity was assessed via electrophoresis on a 1% agarose gel, while the concentration and purity of RNA were determined using a NanoDrop 2000 (OD260/280 ≥ 1.8, OD260/230 ≥ 1.5). RNA integrity was further assessed using an Agilent 2100 Bioanalyzer, with RIN values ≥7.0 deemed acceptable. The library was prepared on the Illumina NovaSeq 6000 platform as 150-bp paired-end reads for sequencing according to the following workflow: mRNA enrichment and fragmentation were performed using the NEBNext^®^ Ultra™ RNA Library Prep Kit; double-stranded cDNA was synthesized, followed by end repair, A-tailing, and sequencing adapter ligation; the library was amplified and enriched via PCR; and the final library was subjected to Qubit quantification and Agilent 2100 quality control before sequencing. A total of ≥6 Gb of sequencing data with ≥97% Q30 base coverage were achieved for each sample. The quality control of the raw reads was performed using Trimmomatic v0.39 to remove adapters, low-quality sequences, and short fragments <50 bp, yielding clean reads. Clean reads were aligned to the reference genome (*Nelumbo nucifera*, NCBI Assembly: GCA_018350525.1, The genome contains 26,685 predicted protein-coding genes) using HISAT2 v2.2.1, achieving >93% alignment efficiency. Gene expression levels were quantified using featureCounts v2.0.3, with expression presented as fragments per kilobase of transcript per million mapped reads (FPKMs). The differential expression analysis was performed using DESeq2 with the following criteria: |log_2_ fold change| ≥ 1 and adjusted p value (FDR) < 0.05. Differentially expressed genes (DEGs) were subjected to GO functional annotation and KEGG pathway enrichment analyses using clusterProfiler, with a significance threshold of an FDR < 0.05. Sequencing and data analysis were conducted by Ling’en Biotechnology Co., Ltd.

## Data Availability

The datasets presented in this study can be found in online repositories. The names of the repository/repositories and accession number(s) can be found in the article/**Supplementary Material**.
