# Supplementary material for: Physiological and molecular mechanisms underlying the quality changes of lotus seeds during different ripening periods
Source: Front Plant Sci. 2026 Apr 16;17:1797105. doi: 10.3389/fpls.2026.1797105 (PMC13128632; doi:10.3389/fpls.2026.1797105)
Supplement: Supplementary file 1 [file DataSheet1.zip › Supplemental Table S1 and Fig S1.docx]

**Table S1. Statistics on Data Volume and Alignment Rate After Quality Control**

| **SampleID** | **Total_Reads** | **Total_Bases** | **Q20%** | **Q30%** | **Mapped_Reads** | **Mapped_Rate(%)** |
| --- | --- | --- | --- | --- | --- | --- |
| A-1 | 51283560 | 7647944794 | 98.96 | 97.99 | 48586044 | 94.74 |
| A-2 | 42966942 | 6414146385 | 99.00 | 98.05 | 41024836 | 95.48 |
| A-3 | 50250068 | 7490898701 | 98.97 | 98.00 | 47300389 | 94.13 |
| B-1 | 44770992 | 6670750263 | 98.85 | 97.78 | 42008621 | 93.83 |
| B-2 | 45410930 | 6771460147 | 98.99 | 98.03 | 42468301 | 93.52 |
| B-3 | 45213482 | 6741197362 | 98.92 | 97.90 | 42487109 | 93.97 |


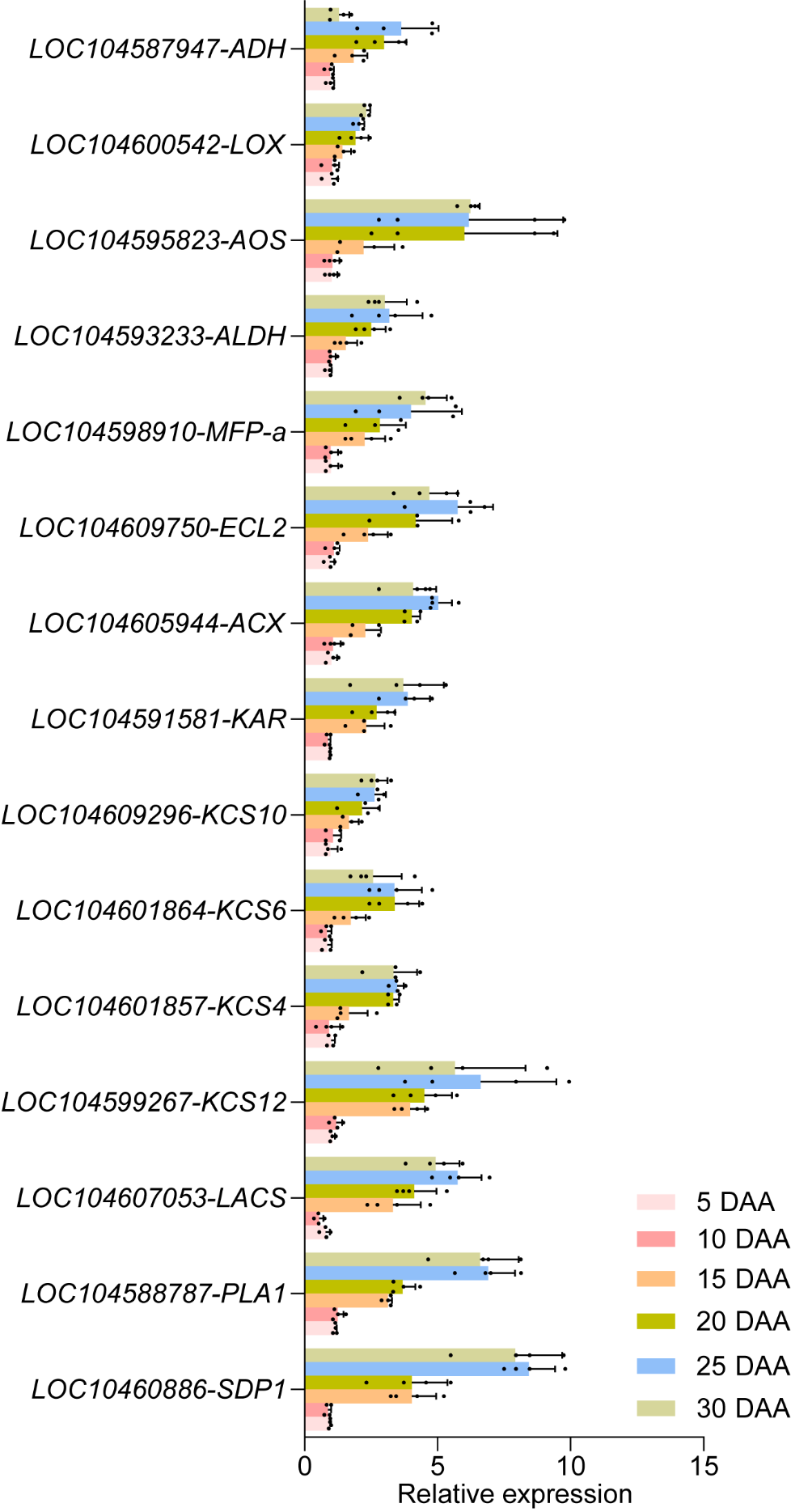


**Figure S1. Expression patterns of genes related to the maturation characteristics of lotus seeds at different developmental stages.** The expression of these lotus seed maturation-related genes at six developmental stages was detected using qRT‒PCR. The data presented are the means and standard errors of four replicates. Different lowercase letters above the bars indicate significant differences between means (*p*< 0.01), as determined using t tests.
